# Ratcheting down the virulence of SARS‐CoV‐2 in the COVID‐19 pandemic

**DOI:** 10.1002/jmv.26067

**Published:** 2020-06-03

**Authors:** Adam Brufsky, Michael T. Lotze

**Affiliations:** ^1^ Department of Hematology‐Oncology, UPMC Hillman Cancer Center, Magee Women's Hospital University of Pittsburgh School of Medicine Pittsburgh Pennsylvania; ^2^ Department of Surgery UPMC Hillman Cancer Center Pittsburgh Pennsylvania

To the Editor,

Muller's ratchet^1^ predicts that when mutation rates are high and a significant proportion of mutations are deleterious, a kind of irreversible ratchet mechanism will gradually decrease the mean fitness of small populations of asexual organisms.^2^ In cell culture, serial passage of genetically bottlenecked vesicular stomatitis virus, which grew quite rapidly on HeLa cells, severely reduced in fitness and virulence within 20 passages in a novel BHK host cell.^2^ Genetic bottlenecks are likely important in intraspecies viral transmission.^3^ RNA‐based viruses are particularly prone to repeated bottleneck events due to high genetic variability and large fluctuations in population size which can lead to dramatic fitness losses or even viral extinction.^4^

Conversely, recombination events with other homologous viruses can lead to emergence of novel properties, particularly during periods of replication and transmission in zoonotic hosts. Coronaviruses (CoVs) utilize a highly conserved replication strategy, making vaccines focusing on this vulnerability highly attractive. Subgenomic RNAs are transcribed by discontinuous transcription regulatory sequences, conserved nucleotides located near the 5′‐end of the genome. Recombination events are thus limited in their ability to propagate in the CoV family.^5^

Although RNA viruses typically have the ability to change rapidly (HIV and HCV) creating so‐called quasi‐species with profound differences in viral replicants due to the infidelity latent in the RNA polymerases, this appears to be less so with proofreading enzymes enabling restitution in the CoVs. Viral fitness is largely determined by the ability to propagate within the host and the host species, some reflecting that the most virulent strains are less successful from a broad host/pathogen perspective given that it ultimately limits viral spread. Genetic bottlenecks are created by interaction of viral variants and their host revealing a narrow range of genotypes successful over time.^6^

Genetic bottlenecks are likely to occur quite frequently with RNA‐based respiratory viruses since respiratory droplets often contain only one to two infectious particles per droplet.^7^ Modeling suggests that such bottlenecks likely drive down the virulence of a pathogen due to stochastic loss of the most virulent phenotypes.^8^

There may be evidence of bottleneck genetic selection with other viruses. In Ebola, mutations arose during the 2013 to 2016 outbreak which were postulated to increase or decrease virulence.^9^ In a macaque model of Ebola, attenuation was suggested, as monkeys infected with Ebola strains isolated from later in the epidemic did have improved survival.^10^

During the severe acute respiratory syndrome (SARS) epidemic of 2003 to 2004, several stable mutations of SARS‐CoV were detected suggestive of bottleneck genetic selection. One of these mutations, a 29‐nucleotide deletion of ORF 8, arose early in the outbreak, was less pathogenic in cell culture, and was postulated to result in a loss of viral fitness due to a founder effect.^11^ Interestingly, multiple stable spike protein nonsynonymous substitutions, among others found in various SARS‐CoV protein coding sequences, were found in the later stages of the SARS outbreak as the substitution rate of the coding sequences slowed through time.^12^

Variation in SARS‐CoV‐2 spike proteins have been described, and one of these stable nonsynonymous substitutions, D614G, predicts for increased probability of protein glycosylation at a canonical NXS/T site at residue 616 of the viral spike protein, and correlates with differences in mortality and possibly transmissibility from coronavirus disease‐2019 (COVID‐19) observed comparing both coasts of the United States.^13^ Additional stable nonsynonymous spike protein mutations of SARS‐CoV‐2, including a stable nonsynonymous R408I variant in the receptor binding domain, predicting for decreased viral binding and decreased virulence, have now been described.^14^, ^15^

Several of these mutations are predicted to increase or decrease protein glycosylation. A model of viral infection postulates that SARS‐CoV‐2 infection of the dendritic cell, the type II pneumocyte of the lung, and the endothelial cell is mediated through the DC/L‐SIGN/ACE2/CD209/CD147 complex found on these cells. Decreased glycosylation of the protein spike of SARS‐CoV‐2 should reduce viral binding to DCL‐SIGN and thus reduce viral uptake by these cells.^16^ This should result in a decrease in viral virulence and possibly attenuation.

There are some clues arising from studies of virulence of various SARS‐CoV‐2 isolates. Viral strains isolated from various patients with COVID‐19 in Wuhan can vary as much as 270‐fold in viral load and cytopathogenicity in Vero E6 culture.^17^ These strains contain a number of stable nonsynonymous coding mutations in the spike protein as well as other viral proteins. A protein interactome analysis suggests a rich source of other possible nonsynonymous SARS‐CoV‐2 protein coding alterations that could plausibly alter the virulence of the virus.^18^

The involvement of Muller's ratchet in the pathogenesis and trajectory of the COVID‐19 pandemic should be explored. Examination of the distribution of nonsynonymous SARS‐COV‐2 mutations in symptomatic vs minimally symptomatic patients with COVID‐19 as the pandemic evolves could help support such involvement. Genomic analysis from SARS‐CoV‐2 isolates from Arizona^19^ and Singapore^20^ reveal possible founder mutation deletions in ORF 7 and ORF 8 consistent with the attenuating 29‐nucleotide deletion in ORF 8 in SARS‐CoV found early in the SARS epidemic.^11^

Muller's Ratchet should also be considered in developing vaccines for the disease, balancing natural decreases in virulence and limiting recombination events.

## CONFLICT OF INTERESTS

The authors declare that there are no conflict of interests.
